# William Jarvie, M.D.S.

**Published:** 1922-02

**Authors:** 


William Jarvie, M.D.S. Dr. Jarvie was born in Manchester, England, July 14, 1841, and died in Mount Clair, N. J., November 16, 1921. Dr. Jarvie began the study of dentistry in New York City and Boston from about 1850. In 1863 he located in Brooklyn, N. Y., with his former preceptor, Dr. A. A. Wheeler. In 1886 he was sent as a delegate to the American Dental Convention and from that time on he has been a familiar and interested member of the profession. He organized the Second District Society of New York, and was a continual member for more than twenty-five years. He held many positions of honor and trust in the profession. He established the "Wm. Jarvie Medal Fund," with a contribution of one thousand dollars, and named G. B. Black, W. B. Miller, E. T. Darby, and similar medals are to be granted each year hereafter. Through his influence $125,000 was given to establish the Dental Department of Columbia University.

Dr. Jarvie was a quiet and dignified gentleman and highly respected by his patients and neighbors. He was a devoted student and practitioner, and the members who became acquainted with him always valued his acquaintance and respected him for his devotion to the profession.

